# Association between *JMJD1A* Expression and Sperm Retrieval in Non-Obstructive Azoospermic Patients

**DOI:** 10.22074/cellj.2018.4409

**Published:** 2017-11-04

**Authors:** Zahra Eelaminejad, Raha Favaedi, Tahereh Modarresi, Marjan Sabbaghian, Mohammad Ali Sadighi Gilani, Maryam Shahhoseini

**Affiliations:** 1 Department of Genetics, Reproductive Biomedicine Research Center, Royan Institute for Reproductive Biomedicine, ACECR, Tehran, Iran; 2 Department of Andrology, Reproductive Biomedicine Research Center, Royan Institute for Reproductive Biomedicine, ACECR, Tehran, Iran; 3 Department of Urology, Shariati Hospital, Tehran University of Medical Sciences, Tehran, Iran

**Keywords:** Azoospermia, *JMJD1A*, Nonobstructive, Sperm Retrieval

## Abstract

Identification of molecular markers which can predict the outcome of sperm retrieval non-invasively in patients with non-obstructive azoospermia (NOA) are valuable in clinical andrology. Jumonji domain-containing 1a (JMJD1A) is a significant epigenetic regulator during spermatogenesis, which plays an important role in the differentiation of post-meiotic germ cells into mature spermatozoa. We therefore aimed to examine the potential association between *JMJD1A* expression and the outcome of sperm retrieval in patients with NOA. Testicular biopsy specimens from 50 NOA patients with either successful sperm retrieval (sperm+, n=22) or failed sperm retrieval (sperm-, n=28) were collected and then examined for *JMJD1A* expression by reverse transcription-quantitative polymerase chain reaction (RT-qPCR). In addition, conventional clinical parameters including luteinizing hormone, follicle-stimulating hormone, testosterone, age, and testicular volume were compared between the two NOA groups. The expression of *JMJD1A* in the sperm+ group was significantly higher than in the sperm- group (P<0.001), however, no significant difference was observed between the two groups in clinical parameters. The receiver operating characteristic (ROC) curve of JMJD1A expression in predicting the sperm retrieval outcome showed a sensitivity of 90.91% and a specificity of 89.29% with significant discriminatory ability between the sperm+ and sperm- groups [area under the ROC curve (AUC)= 0.91]. This study demonstrates a significant association between the expression of *JMJD1A* and the success of sperm recovery in patients with NOA, and thus suggests that *JMJD1A* expression quantification in testicular biopsies may be a valuable biomarker along with conventional parameters in predicting the presence of spermatozoa.

Non-obstructive azoospermia (NOA) is one of the most severe forms of male infertility (1) and accounts for almost 10% of all infertile men (2, 3). Testicular sperm extraction (TESE) in conjunction with intracytoplasmic sperm injection (ICSI) is the treatment of choice for these patients (4, 5). However, the success rate of TESE is low (~ 50%) in these men (6). Microdissection TESE, which is implemented by an operative microscope, is an alternative technique with a higher sperm retrieval rate than conventional TESE. Nevertheless, its success rate ranges from 54 to 64% and is still low (7). On the other hand, failed sperm recovery procedures have economic and emotional burden for the couples (8). Hence, it would be a significant step in clinical andrology to identify molecular markers which can predict the presence of testicular spermatozoa in NOA patients (4, 9, 10). Spermatogenesis is a unique multi-step process that relies on a series of highly controlled mechanisms and functions of several chromatin modifying enzymes (11-14). Jumonji domain containing 1A (JMJD1A, a.k.a KDM3A and JHDM2A), is a known histone H3K9me1/me2 demethylase which functions during spermatogenesis (15, 16). This protein is expressed in meiotic and post-meiotic germ cells with highest levels reported in round spermatids (17) while undetectable in mature spermatozoa, Sertoli cells and Leydig cells (18). JMJD1A regulates expression of post-meiotic genes involved in chromatin remodeling and DNA compaction, including transition nuclear protein (*TNP*) and protamine (*PRM*) genes (17, 18).

This protein also plays a direct role in cytoskeletal rearrangements during spermiogenesis by targeting nonhistone substrates in the cytoplasm (19). Previous studies have indicated that JMJD1A knockout mice are infertile due to several post-meiotic defects including impaired chromatin condensation, defective heterochromatin distribution, cytoskeletal disorders, incomplete acrosome formation and abnormal head morphologies of spermatids (17-19). In addition, we recently showed a defect in the expression of *JMJD1A/JMJD1A* in patients with round spermatid maturation arrest, suggesting its involvement in post-meiotic maturation extends to humans (20). Given the significant role of histone demethylase JMJD1A in differentiation of post-meiotic germ cells into mature spermatozoa (17-20), we aimed to examine the potential association between the *JMJD1A* expression level and the presence of spermatozoa in NOA patients who underwent microdissection TESE.

This study was approved by the Institutional Ethics Committee of the Royan Institute, and written informed consent was obtained from all patients. Testicular biopsies were collected from 50 patients with NOA who underwent sperm retrieval for ICSI. The sperm retrieval was implemented by microdissection TESE according to that described by Schlegel (21). Clinical parameters including luteinizing hormone (LH), follicle-stimulating hormone (FSH), testosterone (T), age, and testicular volume were recorded. After performing microdissection TESE, a small part of each biopsy was immersed in Bouin’s solution for histological analysis according to the approach of McLachlan et al. (22) while the remainder was used for RNA isolation. Based on the histological ﬁndings, the specimens were classified into three groups of hypospermatogenesis (HS, n=9), maturation arrest (MA, n=24), and Sertoli cell only (SCO, n=17) (Fig .1).

Total RNA was purified with TRIzol Reagent (Invitrogen, USA) from the biopsies according to the manufacturer’s protocol. After DNase I treatment, first-strand cDNA was synthesized using the RevertAid™ H Minus First Strand cDNA Synthesis Kit (Fermentas, Germany) as per the manufacturer’s directions. The prepared cDNA samples were then subjected to quantitative polymerase chain reaction (qPCR) on a 7500 Real-Time PCR System (AB Applied Biosystems, USA) using SYBR Green master mix (AB Applied Biosystems, USA) and genespecific primers listed in Table 1 (23). All samples were normalized against the expression of the *β-actin* gene (18). The relative gene expression was analyzed with the 2^-ΔΔCt^ method (24). Statistical analysis was performed with the IBM SPSS statistics version 20 (IBM Corp, USA). Quantitative variables were expressed as mean ± SEM. Statistical comparisons were examined by independent t test and P<0.05 were considered significant. Receiver operating characteristic (ROC) curve was obtained to determine the performance of *JMJD1A* expression in predicting sperm retrieval outcome.

In this study, sperm retrieval was successful in 22 of the 50 studied patients of which 8, 5 and 9 were MA, SCO and HS respectively. The failed sperm retrieval group consisted of 16 patients with MA and 12 patients with SCO. Several parameters were then compared between the successful sperm retrieval (sperm+) and failed sperm retrieval (sperm) NOA groups including *JMJD1A* expression, age, testicular volume, and serum levels of LH, FSH and T (Table 2). The data revealed a significantly higher expression of *JMJD1A* in the sperm+ NOA group (fold-change: 0.098, P<0.001), however, no significant difference was observed for any of the other parameters. Similarly, all parameters were compared among histological sub-groups. Expression of *JMJD1A* was significantly higher in the sperm+ NOA group within the MA (Fold-change:-0.059, P=0.003) and SCO (fold-change:-0.044, P<0.001) sub-groups when compared with the same sub-groups in the sperm- NOA group. Consistently, there were no significant differences in age, testicular volume, and the serum LH, FSH and T levels between the two NOA groups within the MA and SCO sub-groups. This comparison was irrelevant for the HS subgroup since all cases of this subgroup had successful sperm retrieval. Nonetheless, the expression of *JMJD1A* in the HS subgroup was high (1.22 ± 0.1, fold-change: 0.29).

**Fig.1:**
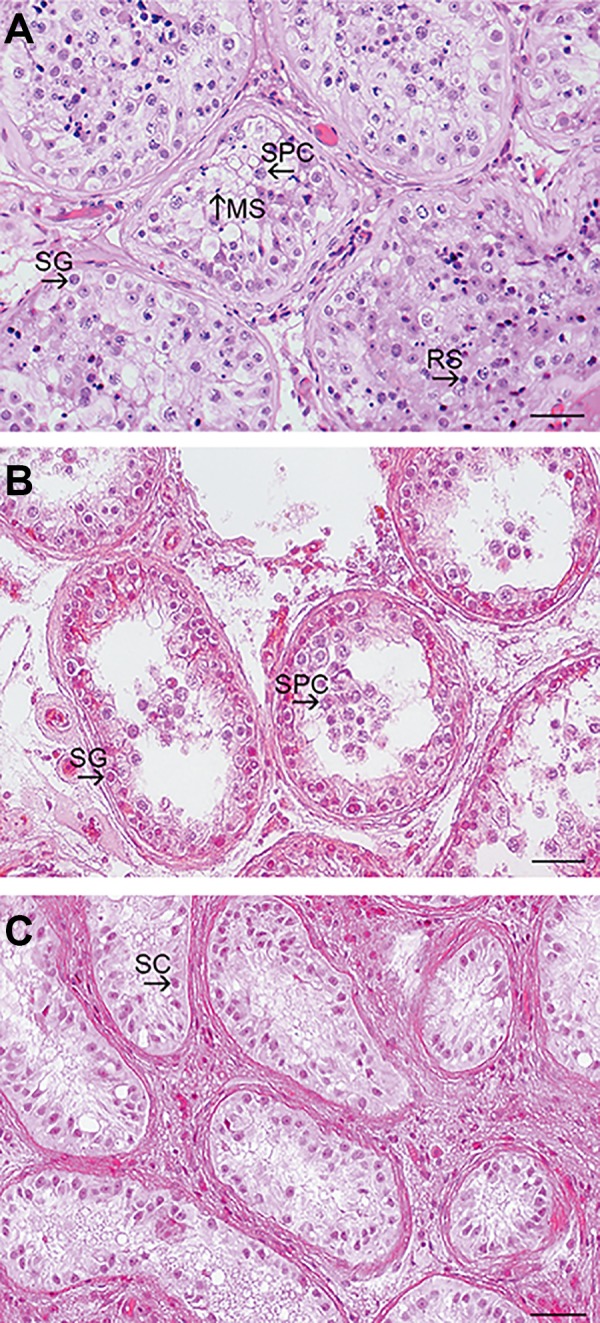
Hematoxylin and Eosin (H&E) staining of histological sections of patients. Specimens were classified into three groups of A. Hypospermatogenesis, B. Maturation arrest, and C. Sertoli cell only. SC; Sertoli cells, SG; Spermatogonia, SPC; Spermatocytes, RS; Round spermatids, and MS; Mature spermatids (scale bar=50 μm).

**Table 1:** Quantitative polymerase chain reaction primers used in this study


Gene	Primer sequencing (5' → 3')	Product size (bp)	Annealing temperature (˚C)

*JMJD1A*	F: CAGTTGCCTAAATGCCGA	111	60
	R: TGAATTGTAACCTCCTGAAGTG		
*ACTB*	F: AGCACAGAGCCTCGCCTT	163	60
	R: CACGATGGAGGGGAAGAC		


**Table 2:** Comparison of all parameters tested in the NOA patients based on the outcome of sperm retrieval


	Group	Number of patients	Age (Y)	Testicular volume (mL)	LH (mIU/ml)	FSH (mIU/ml)	T (ng/ml)	JMJD1A transcript expression

Entire study population								
	Sperm+	22	32.75 ± 1.6	14.01 ± 1.48	7.06 ± 1.39	13.62 ± 3.13	3.97 ± 0.72	1.07 ± 0.08
	Sperm-	28	31.24 ± 0.82	11.42 ± 1.38	5.76 ± 0.91	11.51 ± 1.56	4.91 ± 0.55	0.39 ± 0.06
	P value		0.37	0.21	0.42	0.51	0.31	<0.001^*^
Maturation arrest								
	Sperm+	8	33.5 ± 2.53	14.13 ± 2.3	5.74 ± 1.55	9.79 ± 1.95	4.53 ± 1.33	0.96 ± 0.16
	Sperm-	16	32.3 ± 1.07	10.87 ± 1.5	5.61 ± 1.24	9.82 ± 1.85	5.15 ± 0.75	0.42 ± 0.08
	P value		0.61	0.24	0.96	0.99	0.72	0.003^*^
Sertoli cell only								
	Sperm+	5	30.25 ± 2.25	9.2 ± 2.43	8.17 ± 3.76	22.73 ± 8.27	3.03 ± 0.5	0.97 ± 0.08
	Sperm-	12	29.71 ± 1.15	12.35 ± 2.85	6.05 ± 1.32	14.41 ± 2.63	4.34 ± 0.42	0.34 ± 0.08
	P value		0.82	0.44	0.52	0.23	0.1	<0.001^*^
Hypospermatogenesis								
	Sperm+	9	34.5 ± 3.66	16.95 ± 2.14	7.73 ± 2.8	9.63 ± 3.47	4.33 ± 1.89	1.22 ± 0.1
	Sperm-	0	N/A	N/A	N/A	N/A	N/A	N/A
	P value	N/A	N/A	N/A	N/A	N/A	N/A	N/A


NOA; Non-obstructive azoospermia, LH; Luteinizing hormone, FSH; Follicle-stimulating hormone, T; Testosterone, Sperm+; Patients with successful sperm retrieval, Sperm-; Patients with failed sperm retrieval, N/A; Not applicable, and *; Significant difference, independent t test. Values are mean ± SEM.

The expression level of *JMJD1A* in each patient was presented with respect to the sperm retrieval outcome (Fig .2). The ROC curve analysis of *JMJD1A* transcript level in predicting retrievable sperm (Fig .2) indicated that the optimal cut-off level of *JMJD1A* was 0.74 for the entire study population, 0.80 for the MA subgroup and 0.67 for the SCO subgroup. The area under the ROC curve (AUC) for the entire study population, the MA subgroup and the SCO subgroup were 0.91, 0.84, and 0.95, respectively.

At these cut-off levels, sensitivity, specificity, positive predictive value (PPV), negative predictive value (NPV), positive likelihood ratio (PLR) and negative likelihood ratio (NLR) for predicting the outcome of sperm retrieval were respectively 90.91, 89.29, 86.96, 92.59%, 8.49 and 0.1 for the entire study population, 75, 87.5, 75, 87.5%, 6 and 0.29 for the MA subgroup, and 100, 91.67, 83.33, 100%, 12.005 and 0 for the SCO subgroup. Several parameters are used to predict successful sperm recovery in NOA patients which include hormonal concentration, testicular volume, semen analysis and histological diagnosis, however, their predictive power is low (4, 5, 25). Recently, it has been proposed that the expression of molecular markers involved in spermatogenesis may potentially predict the presence of testicular spermatozoa in men with NOA (4, 9, 10). Several molecular markers to date have been suggested such as ESX1 which was detected in 95.4% of studied samples with residual or complete spermatogenesis (9) with a sensitivity of 80% and a specificity of 74% for residual spermatogenesis (26). In another study, the transcript level of VASA was tested using reverse transcription-quantitative polymerase chain reaction (RT-qPCR) in 52 men with NOA and was shown to be an independent predictive factor for sperm recovery with 87.0% sensitivity and 86.2% specificity (4).

Stahl et al. (5) evaluated the expression of *HSFY* by RTqPCR and suggested its potential as a diagnostic marker for sperm retrieval in NOA patients with sensitivity of 66.7% and specificity of 92.6%. Despite the identification of these markers, the search for identifying more accurate diagnostic markers is still ongoing (4, 9, 10).

**Fig.2:**
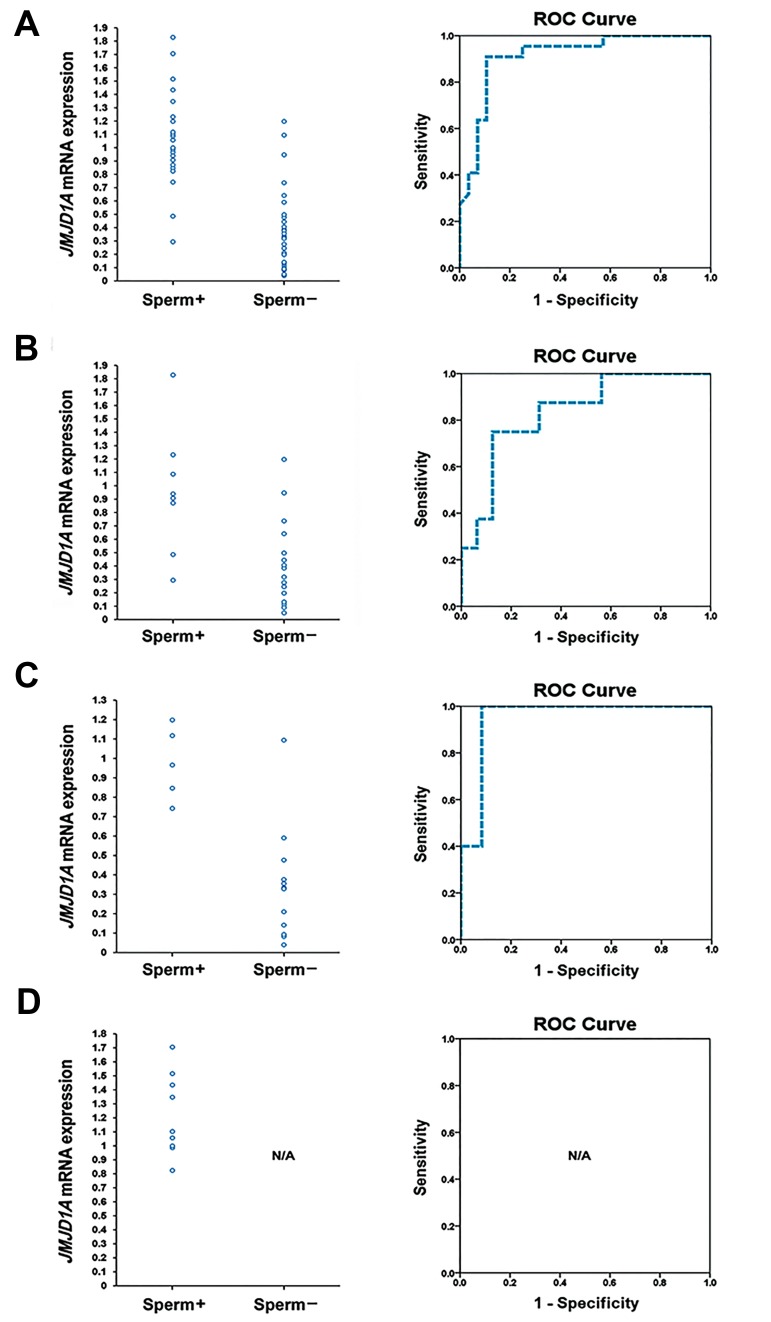
Diagram of *JMJD1A* expression level in each patient based on the outcome of sperm retrieval as well as ROC curve of *JMJD1A* expression in predicting the sperm retrieval outcome. A. Entire study population, B. Maturation arrest sub-group, C. Sertoli cell only sub-group, and D. Hypospermatogenesis subgroup. Sperm+; Patients with successful sperm retrieval, Sperm-; Patients with failed sperm retrieval, ROC; Receiver operating characteristic and N/A; Not applicable.

In the current study, we examined the potential value of histone demethylase JMJD1A as a predictor of successful sperm retrieval in 50 NOA patients. The expression of *JMJD1A* at the transcript level was significantly higher in the sperm+ group than the sperm- group. In addition, the AUC of *JMJD1A* expression for the entire study population was 0.91, indicating the discriminatory power of *JMJD1A* expression in differentiating between sperm+ and sperm- NOA groups. Based on ROC curve analysis, *JMJD1A* expression showed a sensitivity of 90.91% and a specificity of 89.29% with a cut-off level of 0.74. These results are in agreement with those reported by Javadirad et al. (27) and present JMJD1A as a more reliable marker for predicting sperm recovery than the many previously proposed markers. Moreover, JMJD1A not only distinguished the sperm+ group from the sperm- group, but it also discriminated between the two groups when limited to each histological sub-group of NOA. The latter finding is of great importance for the SCO sub-group since it makes it possible to select the patients who are most likely to have occult foci of spermatogenesis. The lack of significant differences in clinical parameters further strengthens the usefulness of this molecular marker.

Considering the diagnostic power of JMJD1A in differentiating the sperm+ group from the sperm− group, quantifying the expression of *JMJD1A* is likely to be useful for infertile men with failed microsurgical testicular sperm extraction (microTESE) for them to decide whether to repeat this surgical procedure. In other words, if the outcome of the first microTESE is unsuccessful and the JMJD1A transcript level in the biopsy specimen is high, it may be valuable to repeat microTESE. However, if the outcome of microTESE is unsuccessful and the JMJD1A transcript level is low, repeating microTESE is probably not worthwhile. In this case, using donated sperm is recommended. Furthermore, given that H3K9me (H3K9me2/3) constitutes a barrier to efficient reprogramming (28), a high level of *JMJD1A* expression could also be an indicator of the sperm quality in terms of their ability to be reprogrammed by the oocyte. Therefore, in the absence of significant *JMJD1A* expression in spermatogenic cells, even if spermatozoa are found in the biopsy, they might be of poor "epigenetic" quality and unable to support a normal development after ICSI.

In conclusion, our study demonstrates a significant association between *JMJD1A* expression and the success of sperm retrieval in patients with NOA. We propose quantifying the expression of *JMJD1A* as a useful adjunct to conventional clinical parameters for predicting the outcome of sperm retrieval in NOA patients. This will likely lead to the selection of patients who are most likely to have sperm and to avoid the repetition of unnecessary surgical procedures. However, for this to be clinically approved, additional studies with much larger sample sizes are needed to independently confirm these findings.
